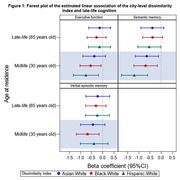# Lifecourse city‐level residential segregation and late life cognitive health in a diverse cohort: Findings from KHANDLE and STAR

**DOI:** 10.1002/alz70860_107195

**Published:** 2025-12-23

**Authors:** Yi Lor, Michelle J Ko, Peter James, Kristen M. George, Hilary L. Colbeth, Chinomnso Okorie, Paola Gilsanz, Rachel A. Whitmer

**Affiliations:** ^1^ University of California, Davis, Davis, CA, USA; ^2^ University of California Davis, Davis, CA, USA; ^3^ University of California Davis Center for Occupational and Environmental Health, Davis, CA, USA; ^4^ Kaiser Permanente Northern California Division of Research, Pleasanton, CA, USA

## Abstract

**Background:**

Residential segregation in the U.S. is a fundamental cause of health disparities contributing to socioeconomic, environmental, and structural differences between neighborhoods. Its role in risk for and resilience to dementia is not well known.

**Method:**

KHANDLE and STAR are harmonized cohorts of 40% Black, 20% White, 17% Asian, and 14% Latinx older adults. Participant self‐reported residence geolocations were linked to interpolated decennial (1970‐2017) census tract population data by race/ethnicity. City‐level residential racial segregation was assessed using the dissimilarity index, linked to participant's mid‐ (age 30) and late‐life (age 65) timepoint. The city‐level dissimilarity index sums, across a city's census tracts, the differences in the ratio of racial/ethnic group's population at the census tract‐level compared to the city‐level for each racial minority groups versus White populations. This number is then divided by 2 and reflects the distribution within a city of a minority group (Asian, Black, or Hispanics) compared to White group on a scale of 0 (complete integration) to 1 (complete segregation). Executive function (EF), verbal episodic memory (VEM), and semantic memory (SM) were collected at study baseline (year 2017‐2018) using the Spanish English Neuropsychological Assessment Scale battery. Linear regression models examined the association of city‐level dissimilarity at separate residence age and domain‐specific cognition, adjusting for age and sex/gender, for the whole sample regardless of race/ethnicity.

**Result:**

Among 2476 participants (average age 72.7; SD = 6.9) years, 62% were female, and 47% had a college education. Higher city‐level dissimilarity (more segregation) at age 30 was associated with lower SM for all groups (Asian‐White: β[95% CI]=‐0.641[‐1.164, ‐0.118], Black‐White: β[95% CI]=‐0.69[‐1.219, ‐0.162], Hispanic‐White: β[95% CI]=‐1.191[‐1.775, ‐0.607]). Higher city‐level Black‐White dissimilarity at midlife was additionally associated with lower VEM (β[95% CI]=‐0.681[‐1.19, ‐0.172]) and Hispanic‐White dissimilarity was associated with lower EF (β[95% CI]=‐0.548[‐1.02, ‐0.076]). At age 65, only higher Black‐White dissimilarity was associated with lower SM (β[95% CI]=‐0.535[‐1.051, ‐0.019]).

**Conclusion:**

Midlife residential segregation measured by the city‐level dissimilarity index is associated with lower late‐life semantic memory, which support evidence in other studies that found higher segregation was associated with lower cognition. Early adulthood may be a sensitive period where residential segregation is especially impactful on late‐life cognition.